# Supplementary material for: Exploring structural dynamics of a membrane protein by combining bioorthogonal chemistry and cysteine mutagenesis
Source: eLife. 2019 Nov 12;8:e50776. doi: 10.7554/eLife.50776 (PMC6850778; doi:10.7554/eLife.50776)
Supplement: Supplementary file 1. [file elife-50776-supp1.docx]

**Table S1. Dibenzocyclooctyne (DBCO)-conjugated fluorophores**

| 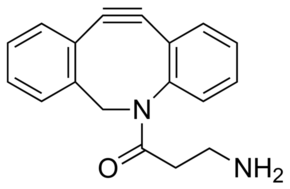**Name** | **Excitation/Emission**  **(nm)** | **Extinction Coefficient**  **(M^-1^cm^-1^)** | **Molecular weight**  **(Da)** | **Solubility** |
| --- | --- | --- | --- | --- |
| AF405 DBCO | 404/424 | 35000 | 774.79 | Water, DMSO, DMF |
| AF430 DBCO | 430/539 | 15000 | 761.81 | Water, DMSO, DMF |
| AF488 DBCO | 494/517 | 73000 | 995.18 | Water, DMSO, DMF |
| Carboxyrhodamine 110 DBCO | 501/523 | 74000 | 880.96 | DMSO, DMF |
| AF532 DBCO | 530/555 | 81000 | 825 | Water, DMSO, DMF |
| TAMRA DBCO | 548/562 | 92000 | 936.09 | DMSO, DMF |
| AF546 DBCO | 554/570 | 112000 | 1104.15 | Water, DMSO, DMF |
| AF555 DBCO | 555/572 | 155000 | 1131.36 | Water, DMSO, DMF |
| Cy3 DBCO | 553/569 | 150000 | 983.18 | Water, DMSO, DMF |
| AF568 DBCO | 578/602 | 88000 | 953.04 | Water, DMSO, DMF |
| AF594 DBCO | 590/617 | 92000 | 1082.31 | Water, DMSO, DMF |
| AF647 DBCO | 648/671 | 270000 | 1131.36 | Water, DMSO, DMF |
| Cy5 DBCO | 649/671 | 250000 | 1009.62 | Water, DMSO, DMF |
| Cy5.5 DBCO | 678/694 | 190000 | 1175.37 | Water, DMSO, DMF |
| AF750 DBCO / Cy7 DBCO | 753/775 | 255000 | 1231.47 | Water, DMSO, DMF |
| MB488 DBCO | 501/524 | 75000 | 913.94 | Water, DMSO |
| MB543 DBCO | 543/563 | 105000 | 1074.34 | Water, DMSO, DMF |
| MB594 DBCO | 601/623 | 110000 | 1102.26 | Water, DMSO, DMF |
| MB660 R DBCO | 665/690 | 92000 | 1003.19 | Water, DMSO, DMF |

<https://clickchemistrytools.com/product-category/fluorescent-dyes/cu-free-click-chemistry/> - All these fluorophores are charged and membrane impermeable.
