# Supplementary material for: Exploring structural dynamics of a membrane protein by combining bioorthogonal chemistry and cysteine mutagenesis
Source: eLife. 2019 Nov 12;8:e50776. doi: 10.7554/eLife.50776 (PMC6850778; doi:10.7554/eLife.50776)
Supplement: Supplementary file 2. [file elife-50776-supp2.docx]

**Table S2. Bicyclononyne (BCN)–conjugated fluorophores**

| 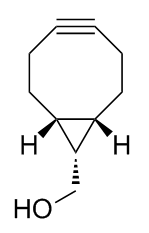**Name** | **Excitation/Emission**  **(nm)** | **Extinction Coefficient**  **(M^-1^cm^-1^)** | **Molecular weight**  **(Da)** | **Solubility** |
| --- | --- | --- | --- | --- |
| CF405S BCN | 404/431 | 33000 | 788 | Water, DMSO |
| CF405M BCN | 408/452 | 41000 | 720 | Water, DMSO |
| CF488A BCN | 490/515 | 70000 | 1132 | Water, DMSO |
| CF568 BCN | 562/583 | 100000 | 932 | Water, DMSO |
| CF594 BCN | 593/614 | 115000 | 947 | Water, DMSO |
| CF640R | 642/662 | 105000 | 1050 | Water, DMSO |
| CF680R | 680/701 | 140000 | 1130 | Water, DMSO |

<https://biotium.com/product/cf-dye-bcn/-> - All these fluorophores are membrane impermeable.
