# Supplementary material for: Exploring structural dynamics of a membrane protein by combining bioorthogonal chemistry and cysteine mutagenesis
Source: eLife. 2019 Nov 12;8:e50776. doi: 10.7554/eLife.50776 (PMC6850778; doi:10.7554/eLife.50776)
Supplement: Supplementary file 3. [file elife-50776-supp3.docx]

**Table S3. Prevalence and location of methionine and cysteine residues in a selection of membrane proteins previously studied with voltage-clamp fluorometry.**

| **Protein** | | **Methionine** | **Cysteine** | **Total** |
| --- | --- | --- | --- | --- |
| **Shaker / KCNAS_DROME**  GenBank : AAA28417.1  Uniprot : P08510 | Extracellular | 2 | 0 | 90 |
|  | Membrane | 2 | 3 | 145 |
|  | Cytoplasmic | 8 | 4 | 420 |
|  | Total | 12 | 7 | 655 |
| hKv7.1/ KCNQ1_HUMAN Genbank : AAC51776.1  Uniprot : P51787 | Extracellular | 0 | 0 | 37 |
|  | Membrane | 3 | 4 | 149 |
|  | Cytoplasmic | 7 | 5 | 490 |
|  | Total | 10 | 9 | 676 |
| **hERG / KCNH2_HUMAN**  GenBank : AAA62473.1  Uniprot : Q12809 | Extracellular | 2 | 2 | 79 |
|  | Membrane | 5 | 3 | 147 |
|  | Cytoplasmic | 17 | 19 | 933 |
|  | Total | 24 | 24 | 1159 |
| EAG/KCNAE_DROME Genbank : AAA28495  Uniprot : Q02280 | Extracellular | 2 | 0 | 80 |
|  | Membrane | 5 | 7 | 163 |
|  | Cytoplasmic | 22 | 13 | 931 |
|  | Total | 29 | 20 | 1174 |
| spHCN/ IcH_ STRPU Genbank : CAA76493 (Sea Urchin)  UniProtKB - O60741 (HCN1_HUMAN) | Extracellular | 2 | 0 | 64 |
|  | Membrane | 8 | 8 | 151 |
|  | Cytoplasmic | 14 | 6 | 552 |
|  | Total | 24 | 14 | 767 |
| **hBK / KCMA1_HUMAN**  GenBank: AAB65837.1 Uniprot : Q12791 | Extracellular | 3 | 2 | 63 |
|  | Membrane | 6 | 1 | 170 |
|  | Cytoplasmic | 24 | 32 | 938 |
|  | Total | 33 | 35 | 1171 |
| rNaV1.4 / SCN4A_RATGenBank : AAA41682.1 Uniprot : P15390 | Extracellular | 12 | 12 | 367 |
|  | Membrane | 29 | 11 | 552 |
|  | Cytoplasmic | 25 | 16 | 921 |
|  | Total | 66 | 39 | 1840 |
| **hNaV1.5 / SCN5A_HUMAN**  GenBank : AAA58644.1  Uniprot : Q14524 | Extracellular | 9 | 10 | 339 |
|  | Membrane | 31 | 11 | 554 |
|  | Cytoplasmic | 29 | 21 | 1123 |
|  | Total | 69 | 42 | 2016 |
| **hCaV1.2 / CAC1C_HUMAN**  GenBank : AAA17030.1  Uniprot : Q13936 | Extracellular | 7 | 12 | 370 |
|  | Membrane | 23 | 11 | 575 |
|  | Cytoplasmic | 24 | 21 | 1276 |
|  | Total | 54 | 44 | 2221 |
| **hGlyR1 / GLRA1_HUMAN**  GenBank : AAH74980.1  Uniprot : P23415 | Extracellular | 10 | 5 | 272 |
|  | Membrane | 5 | 1 | 85 |
|  | Cytoplasmic | 4 | 1 | 100 |
|  | Total | 19 | 7 | 457 |
| **ELIC / ELIC_DICCH**  Uniprot : P0C7B7 | Extracellular | 2 | 0 | 213 |
|  | Membrane | 2 | 1 | 84 |
|  | Cytoplasmic | 1 | 1 | 24 |
|  | Total | 5 | 2 | 321 |
| **rP2X1 / P2RX1_RAT**  NCBI : NP_037129.1  Uniprot : P47824 | Extracellular | 3 | 10 | 288 |
|  | Membrane | 0 | 1 | 42 |
|  | Cytoplasmic | 4 | 0 | 69 |
|  | Total | 7 | 11 | 399 |
| **mASIC1/ ASIC1_MOUSE**  Genbank : BAD97849.1  Uniprot : Q6NXK8 | Extracellular | 10 | 16 | 356 |
|  | Membrane | 1 | 3 | 51 |
|  | Cytoplasmic | 2 | 4 | 119 |
|  | Total | 13 | 23 | 526 |
| **ciHV1/ HVCN1_CIOIN**  Genbank : BAE94277.1  Uniprot : Q1JV40 | Extracellular | 0 | 0 | 25 |
|  | Membrane | 1 | 0 | 86 |
|  | Cytoplasmic | 8 | 3 | 231 |
|  | Total | 9 | 3 | 342 |
| **hSGLT1 / SC5A1_HUMAN**  GenBank: AAA60320.1  Uniprot : P13866 | Extracellular | 9 | 9 | 269 |
|  | Membrane | 14 | 5 | 312 |
|  | Cytoplasmic | 4 | 1 | 83 |
|  | Total | 27 | 15 | 664 |
| **hSERT /SC6A4_HUMAN**  GenBank: AAA35492.1  UniProtKB - P31645 | Extracellular | 3 | 2 | 164 |
|  | Membrane | 7 | 9 | 258 |
|  | Cytoplasmic | 2 | 7 | 208 |
|  | Total | 12 | 18 | 630 |
| flounder NaPi-IIb/ NPT2B GenBank : AAB16821  [Uniprot : O95436 (Human)](https://www.uniprot.org/uniprot/O95436) | Extracellular | 4 | 4 | 203 |
|  | Membrane | 3 | 6 | 168 |
|  | Cytoplasmic | 14 | 9 | 265 |
|  | Total | 21 | 19 | 636 |
| **Xenopus NaK-ATPase-α1 /**  **AT1A1_XENLA**  GenBank: AAA19022.1  Uniprot : Q92123 | Extracellular | 3 | 3 | 112 |
|  | Membrane | 4 | 5 | 204 |
|  | Cytoplasmic | 19 | 15 | 709 |
|  | Total | 26 | 23 | 1025 |
| **Xenopus NaK-ATPase-β3/**  **AT1B3_XENLA**  GenBank: AAA49650.1  Uniprot : P21188 | Extracellular | 6 | 6 | 221 |
|  | Membrane | 0 | 0 | 21 |
|  | Cytoplasmic | 2 | 0 | 35 |
|  | Total | 8 | 6 | 277 |
| **rGAT-1 / SC6A1_RAT**  Genbank : AAA63487.1  Uniprot : P23978 | Extracellular | 6 | 3 | 175 |
|  | Membrane | 9 | 8 | 238 |
|  | Cytoplasmic | 5 | 3 | 186 |
|  | Total | 20 | 14 | 599 |
